# Effects of non-modifiable risk factors of Alzheimer’s disease on intracortical myelin content

**DOI:** 10.1186/s13195-022-01152-y

**Published:** 2022-12-31

**Authors:** Marina Fernandez-Alvarez, Mercedes Atienza, Jose L. Cantero

**Affiliations:** 1 Laboratory of Functional Neuroscience, Pablo de Olavide University, Ctra. de Utrera Km 1, 41013 Seville, Spain; 2 CIBERNED, Network Center for Biomedical Research in Neurodegenerative Diseases, Madrid, Spain

**Keywords:** Aging, Alzheimer’s disease, APOE4, Cortical microstructure integrity, Family history, Intracortical myelin, Functional connectivity, T1w/T2w ratio maps

## Abstract

**Background:**

Non-modifiable risk factors of Alzheimer’s disease (AD) have lifelong effects on cortical integrity that could be mitigated if identified at early stages. However, it remains unknown whether cortical microstructure is affected in older individuals with non-modifiable AD risk factors and whether altered cortical tissue integrity produces abnormalities in brain functional networks in this AD-risk population.

**Methods:**

Using relative T1w/T2w (rT1w/T2w) ratio maps, we have compared tissue integrity of normal-appearing cortical GM between controls and cognitively normal older adults with either APOE4 (*N* = 50), with a first-degree family history (FH) of AD (*N* = 52), or with the co-occurrence of both AD risk factors (APOE4+FH) (*N* = 35). Additionally, individuals with only one risk factor (APOE4 or FH) were combined into one group (*N* = 102) and compared with controls. The same number of controls matched in age, sex, and years of education was employed for each of these comparisons. Group differences in resting state functional connectivity (rs-FC) patterns were also investigated, using as FC seeds those cortical regions showing significant changes in rT1w/T2w ratios.

**Results:**

Overall, individuals with non-modifiable AD risk factors exhibited significant variations in rT1w/T2w ratios compared to controls, being APOE4 and APOE4+FH at opposite ends of a continuum. The co-occurrence of APOE4 and FH was further accompanied by altered patterns of rs-FC.

**Conclusions:**

These findings may have practical implications for early detection of cortical abnormalities in older populations with APOE4 and/or FH of AD and open new avenues to monitor changes in cortical tissue integrity associated with non-modifiable AD risk factors.

**Supplementary Information:**

The online version contains supplementary material available at 10.1186/s13195-022-01152-y.

## Background

Due to the rapidly aging population, there is an urgent need for identification of individuals at increased risk of developing Alzheimer’s disease (AD), the most prevalent neurodegenerative condition affecting around 55 million people worldwide [1]. As pharmacological strategies targeting neuropathological hallmarks of AD have proven to be clinically ineffective, identifying AD risk factors appears to be crucial for tailoring interventions aimed to prevent or delay disease onset. Apart from advanced age, having a first-degree family history (FH) of late-onset sporadic AD [2, 3] and carrying the Apolipoprotein E ɛ4 allele (APOE4) [4, 5] are well-documented non-modifiable risk factors for the development of AD and thus represent target populations to promote strategies aimed at ameliorating their consequences.

Myelin plays a pivotal role in neuronal communication by enhancing the conduction speed of action potentials and supporting precise temporal relationships among neurons, which has relevance for neural coding, neuronal integration and synaptic plasticity [6]. Thus, myelin loss results in alterations of spike timing that in turn could influence neural circuit dynamics and, ultimately, leads to aging-related cognitive dysfunctions and a variety of neurodegenerative disorders [7]. Particularly, the neocortex is one of the most plastic and vulnerable late-myelinating structures [8] susceptible to age-related decline [9], and cortical myelination has shown to be sensitive to experience-dependent neuronal activity [10].

Oligodendrocytes, the cells responsible for myelination of neuronal axons, require high cholesterol content for their maturation [11]. In this context, the APOE4 protein has shown reduced efficiency to transport lipids between neurons compared to the more common APOE3 [12]. Consequently, the number of oligodendrocytes is lower in APOE4 brains across the AD continuum regardless of their Braak stage, amyloid deposition, and neuronal loss, suggesting that disruption of myelination in APOE4 carriers may represent a specific pathology in the aging brain [13]. Likewise, cognitively normal older adults with parental FH of AD have shown changes in microstructural white matter (WM) integrity [14, 15], accompanying the greater cerebral amyloid-beta (Aβ) burden observed in this AD-risk population [16]. However, there is lack of evidence that carrying the APOE4 genotype and/or having a first-degree FH of AD has an impact on the tissue integrity of the normal-appearing cortical gray matter (GM), which also contains a considerable amount of myelinated axons [17].

Converging evidence has revealed that subtle changes in myelin have substantial effects on temporal synchrony and neuronal network function [18–20]. Accordingly, previous research has revealed persistent defects of prepulse inhibition of the startle response in mutant mice with a subtle hypomyelination phenotype [21]. In humans, these changes are particularly evident in unimodal areas [22] and mainly affect long-range functional connections [23]. Remarkably, the relationship between cortical tissue integrity and rs-FC patterns has shown to be modulated by higher plasma levels of neurofilament light chain in cognitively normal older adults [24], likely accounting for aberrant patterns of brain functional connectivity observed in individuals at AD risk [25–27]. This hypothesis has not been yet investigated and may expand our knowledge on structural determinants of cortical network dysfunctions in older adults with non-modifiable risk factors for AD.

In this study, we have examined the hypothesis that carrying APOE4 and/or having a first-degree FH of AD leads to changes in cortical T1w/T2w ratio maps. As variations in cortical microstructure have been further associated with changes in rs-FC [22–24], we predict that cortical regions showing significant group differences in rT1w/T2w ratios support abnormal rs-FC patterns in individuals with non-modifiable risk factors of AD. As the co-occurrence of APOE4 and a FH of AD has shown synergistic effects on Aβ deposition and cerebral glucose metabolism [28], we further expect collective effects of both AD risk factors on rT1w/T2w ratio levels and rs-FC patterns.

## Methods

### Subjects

Three hundred eighty-seven cognitively normal older adults participated in the study. They were recruited from senior citizen’s associations, health-screening programs, and hospital outpatient services. All of them underwent neurological and neuropsychological assessment to discard the presence of dementia and/or objective cognitive impairment. Individuals with medical conditions that affect brain structure or function (e.g., cerebrovascular disease, epilepsy, head trauma, history of neurodevelopmental disease, alcohol abuse, hydrocephalus, and/or intracranial mass) were not included in the study. Participants met the following criteria: (i) normal global cognitive status in the Mini-Mental State Examination (scores ≥ 26); (ii) normal cognitive performance in the neuropsychological tests relative to appropriate reference values for age and education level; (iii) global score of 0 (no dementia) in the Clinical Dementia Rating; (iv) functional independence as assessed by the Spanish version of the Interview for Deterioration in Daily Living Activities [29]; (v) scores ≤ 5 (no depression) in the short form of the Geriatric Depression Scale [30]; and (vi) not be taking medications that affected cognition, sleep, renal, and/or hepatic function. All participants gave informed consent to the experimental protocol approved by the Ethical Committee for Clinical Research of the Junta de Andalucía according to the principles outlined in the Declaration of Helsinki.

From this sample, 50 individuals were APOE4 carriers (5 homozygous for the ɛ4 allele), 52 had a first-degree FH of late-onset sporadic AD, and 35 showed the co-occurrence of both AD risk factors (APOE4+FH; 1 homozygous for the ɛ4 allele). For each of these groups, we selected the same number of controls matched in age, sex, and years of education. A control subject was defined as a cognitively normal older adult, non-APOE4 carrier without FH of AD (neither first- nor second-degree relatives). The characteristics of the sample are detailed in Table 1.

**Table 1:** Demographic and cognitive characteristics of the sample

	Controls	APOE4	Controls	FH	Controls	APOE4+FH	Controls	APOE4 or FH
Age	67.8 ± 6.1	67.8 ± 6.1	67.2 ± 4.8	67.2 ± 4.8	67.5 ± 5.4	67.5 ± 5.4	66.9 ± 5.5	66.9 ± 5.5
Sex (F/M)	28/22	28/22	30/22	30/22	16/19	16/19	58/44	58/44
Education years	10.4 ± 5.1	10.4 ± 5.1	10.8 ± 5	10.8 ± 5	10.4 ± 4.8	10.4 ± 4.8	10.6 ± 5	10.6 ± 5
ApoE4	0	50	0	0	0	35	0	50
FH	0	0	0	52	0	35	0	52
CDR	0	0	0	0	0	0	0	0
MMSE	28.6 ± 1.1	29.1 ± 1.1	29 ± 1	29.1 ± 1	28.9 ± 1.1	28.8 ± 1.2	28.8 ± 1.1	28.9 ± 1
Memory Binding Test								
Total free recall	17.3 ± 4.2	17.1 ± 4.1	17.8 ± 4.7	17.1 ± 3.2	19 ± 4.2	18.2 ± 5.2	17.9 ± 4.4	17.2 ± 4
Total delayed free recall	18.2 ± 3.6	18 ± 5.8	17.2 ± 4.7	17.3 ± 5.1	19.1 ± 3.6	20 ± 6.7	18 ± 4.1	17.6 ± 5.6
Total delayed paired recall	26.5 ± 3.3	26.1 ± 4	24.4 ± 5.5	23.9 ± 3.5	27.2 ± 3.1	26.4 ± 5.7	25.7 ± 4.4	24 ± 4
Boston Naming Test	12.6 ± 2.1	12.4 ± 2.4	12.7 ± 1.8	12.9 ± 1.8	12.9 ± 1.6	12.4 ± 1.2	12.7 ± 1.9	12.7 ± 2
Trail Making Test-A (seconds)	43.9 ± 15.6	43.6 ± 21.3	38.1 ± 11.1	41.3 ± 20.2	41.1 ± 15.8	40.2 ± 15.6	41.2 ± 14.2	41.4 ± 19.6
Trail Making Test-B (seconds)	122 ± 61.8	120.1 ± 65.8	115.2 ± 65.5	112.2 ± 55.3	110.4 ± 51.5	106.5 ± 57.1	117 ± 60.5	114 ± 60
Tower of London (seconds)	364.2 ± 147.4	366.4 ± 113.3	367.8 ± 124.3	371.2 ± 149.6	377.1 ± 119.6	382 ± 127.3	369.2 ± 130.8	373 ± 133.5

Results are expressed as mean ± SD. *F/M* females/males, *FH* first-degree family history of AD, *CDR* Clinical Dementia Rating, *MMSE* Mini Mental State Examination

### Neuropsychological assessment

All participants completed a neuropsychological assessment that included the following tests: Mini-Mental State Examination (MMSE), the Spanish version of the Memory Binding Test [31], the short form of the Boston Naming Test (BNT), Trail Making Test (forms A and B), and the Tower of London.

### MRI acquisition

Images were acquired on a 3T Philips Ingenia MRI scanner using a 32-channel receive-only radio-frequency (RF) head coil and a transmit RF body coil (Philips, Best, Netherlands). The following MRI sequences were acquired in the same session: (i) 3D T1-weighted (T1w) magnetization prepared rapid gradient echo (MPRAGE) in the sagittal plane: repetition time (TR)/echo time (TE) = 2600 ms/4.7 ms, flip angle (FA) = 9°, acquisition matrix = 384 × 384, voxel resolution in acquisition = 0.65 mm^3^ isotropic, resulting in 282 slices without gap between adjacent slices; (ii) 3D T2w VISTA Turbo Spin Echo scan in the sagittal plane: TR/TE: 2500 ms/251 ms, FA = 90°, acquisition matrix = 384 × 384 mm, voxel resolution in acquisition = 0.65 mm^3^ isotropic, resulting in 282 slices without gap between adjacent slices; and (iii) T2w Fast Field Echo images using a blood-oxygen-level-dependent (BOLD) sensitive single-shot echo-planar imaging (EPI) sequence in the axial plane: TR/TE: 2000 ms/30 ms, FA = 80°, acquisition matrix = 80 × 80 mm, voxel resolution in acquisition = 3 mm^3^ isotropic, resulting in 35 slices acquired in posterior to anterior phase-encoding direction with 1 mm of gap between adjacent slices. To allow for optimal B1 shimming, a B1 calibration scan was applied before starting the EPI sequence. We acquired 250 EPI scans preceded by 4 dummy volumes to allow time for equilibrium in the spin excitation. Before starting the acquisition of the EPI sequence, participants were asked to remain still and keep their eyes closed without falling sleep. Pulse and respiratory rates were recorded using the scanner’s built-in pulse oximeter placed on the left-hand index finger and a pneumatic respiratory belt strapped around the upper abdomen, respectively. Brain images were visually examined after each MRI sequence; they were repeated if artifacts were identified. All participants underwent the same protocol in the same MRI scanner at the research MRI facility located at Pablo de Olavide University. The signal-to-noise ratio (SNR) was computed for each MRI sequence (i.e., T1w, T2w, and EPI) to confirm that this parameter did not differ among groups. Figure 1 in the Supplementary Material includes the SNR of each sequence for a representative subject of each group.

### Structural MRI preprocessing and T1w/T2w ratio map generation

T1w scans were preprocessed using Freesurfer v6.0 (https://surfer.nmr.mgh.harvard.edu/). The Freesurfer’s pipeline included brain extraction, automated tissue segmentation, generation of WM and pial surfaces, correction of surface topology and inflation, co-registration, and projection of cortical surfaces to a sphere for the purpose of establishing a surface-based coordinate system [32]. Pial surface misplacements and erroneous WM segmentation were manually corrected on a slice-by-slice basis by one experienced technician. T2w images were registered to T1w images with *bbregister* using a trilinear interpolation method and a boundary-based cost function constrained to 6 degrees of freedom [33].

Individual T1w/T2w ratio volumes were sampled at the halfway between the WM and GM surfaces, resulting in midthickness surface maps of the T1w/T2w ratio. To mitigate contamination of cortical GM intensity by intensities of WM and cerebrospinal fluid (CSF), the tissue fraction effect was corrected in individual T1w/T2w ratio maps using the geometric transfer matrix-derived region-based voxel-wise method implemented in PETsurfer [34]. Finally, individual T1w/T2w ratio maps were projected onto the average cortical surface of each group, Z-transformed across vertices within each subject, and smoothed using non-linear spherical wavelet-based denoising schemes [35]. All processing steps were visually checked for quality assurance. The Z transformation applied to individual T1w/T2w ratio maps allowed us to determine how relatively different a region is compared to the mean cortical microstructure for each individual. This index was referred as the relative T1w/T2w (rT1w/T2w) ratio.

### Functional MRI preprocessing and rs-FC analysis

rs-fMRI data were preprocessed using AFNI functions (https://afni.nimh.nih.gov/afni), version AFNI_20.3.01. For each participant, high-frequency spikes were eliminated (*3dDespike*), time-locked cardiac (measured by pulse oximeter) and respiratory motion artifacts on brain BOLD signals were minimized using RETROICOR [36], time differences in slice-acquisition were corrected (*3dTshift*), EPI scans were aligned using rigid body motion correction and the first volume as reference (*3dVolreg*), and aligned EPI scans were co-registered to their corresponding T1w volumes (*align_epi_anat.py;* cost function: lpc+ZZ).

Dynamics were removed provided that more than 5% of voxels exhibited signal intensities that deviated from the median absolute deviation of time series (*3dToutcount*) and/or when the Euclidean norm (*enorm*) threshold exceeded 0.3 mm in head motion. None of the participants showed more than 20% of artifactual dynamics after applying censoring. Simultaneous regression was further applied to minimize the impact of non-neuronal fluctuations on the rs-fMRI signal (*3dTproject*). Nuisance regressors included the following: (i) six head motion parameters (3 translational and 3 rotational) derived from the EPI scan alignment along with their first-order derivatives, (ii) time series of mean total WM/CSF signal intensity, and (iii) cardiac and respiratory fluctuations plus their derivatives to mitigate effects of extracerebral physiological artifacts on brain BOLD signals.

Preprocessed rs-fMRI scans were projected onto the 5th order icosahedral tessellation of the average cortical surface. Cortical regions showing significant group differences in rT1w/T2w ratios were used as seeds for rs-FC analyses. Surface-based rs-FC seed to whole cortex maps were obtained using the Fisher’s *z*-transform of the corresponding Pearson’s correlation coefficients.

### Sample size estimation

To estimate the sample size, we performed power analysis with the G*Power software (v3.1.9.6) (https://www.psychologie.hhu.de/arbeitsgruppen/allgemeine-psychologie-und-arbeitspsychologie/gpower.html). Given the lack of evidence linking non-modifiable risk factors of AD to changes in T1w/T2w ratio maps and/or rs-FC patterns, an a priori (prospective) power analysis (fixed model, R2 deviation from zero) was performed to achieve statistical power of 80% with a significance level of 0.05 and an overall Cohen’s effect size (*f*^2^) ranging from 0.1 to 0.25. To detect an overall effect size of 0.1, we would require 113 and 133 participants for the additive (3 predictors) and interactive models (5 predictors), respectively. As different sample sizes were employed for assessing group differences in rT1w/T2w ratio, our statistical approach is not expected to identify overall effect sizes smaller than 0.11 and 0.16, respectively, in the case of additive models, and 0.13 and 0.20 in the case of interaction models.

### Statistical analysis

Group differences (i.e., controls versus APOE4; controls versus FH; controls versus APOE4+FH; controls versus APOE4 or FH; APOE4 versus APOE4+FH; and FH versus APOE4+FH) in demographic, cognitive variables, and SNR for each MRI sequence were assessed with two sample *t*-tests using SPSS v22 (SPSS Inc. Chicago, IL).

We applied whole-cortex, vertex-wise analyses of covariance (ANCOVAs) to assess group differences in rT1w/T2w ratio levels, including age and sex as covariates of no interest. The results were corrected for multiple comparisons using a hierarchical statistical model that first controls the family-wise error rate at the cluster level by applying random field theory over smoothed statistical maps (*p*_vertex_ < 0.001, *p*_cluster_ < 0.05), and next controls the false discovery rate at the vertex level within each cluster (*P* < 0.05) over unsmoothed statistical maps [37]. Peaks of clusters that survived correction for multiple comparisons were employed to determine the anatomical location of significant changes using the Desikan-Killiany atlas [38].

Using as FC seeds those cortical regions showing significant group differences in rT1w/T2w ratio levels, we next assessed whole-cortex, vertex-wise group differences in the relationship between rs-FC patterns and mean values of cortical regions showing significant group differences in the T1w/T2w ratio. For this, we first applied one-sample *t* tests in each group to minimize the effect of spurious functional connections between cortical regions. Significant positive functional connections of the two groups were combined to assess group × T1w/T2w ratio interactions adjusted by age and sex.

To determine the effect size, we computed the Cohen’s *f*^2^ for each additive and interactive model [39].

## Results

### Group differences in demographic variables and cognitive function

Table 1 summarizes demographic data and results of cognitive tests for each group. No significant group differences were found in any of these measures. The SNR of each MRI sequence was also statistically comparable between controls and AD risk groups.

### Effects of non-modifiable AD risk factors on T1w/T2w ratio maps

**Fig. 1:**
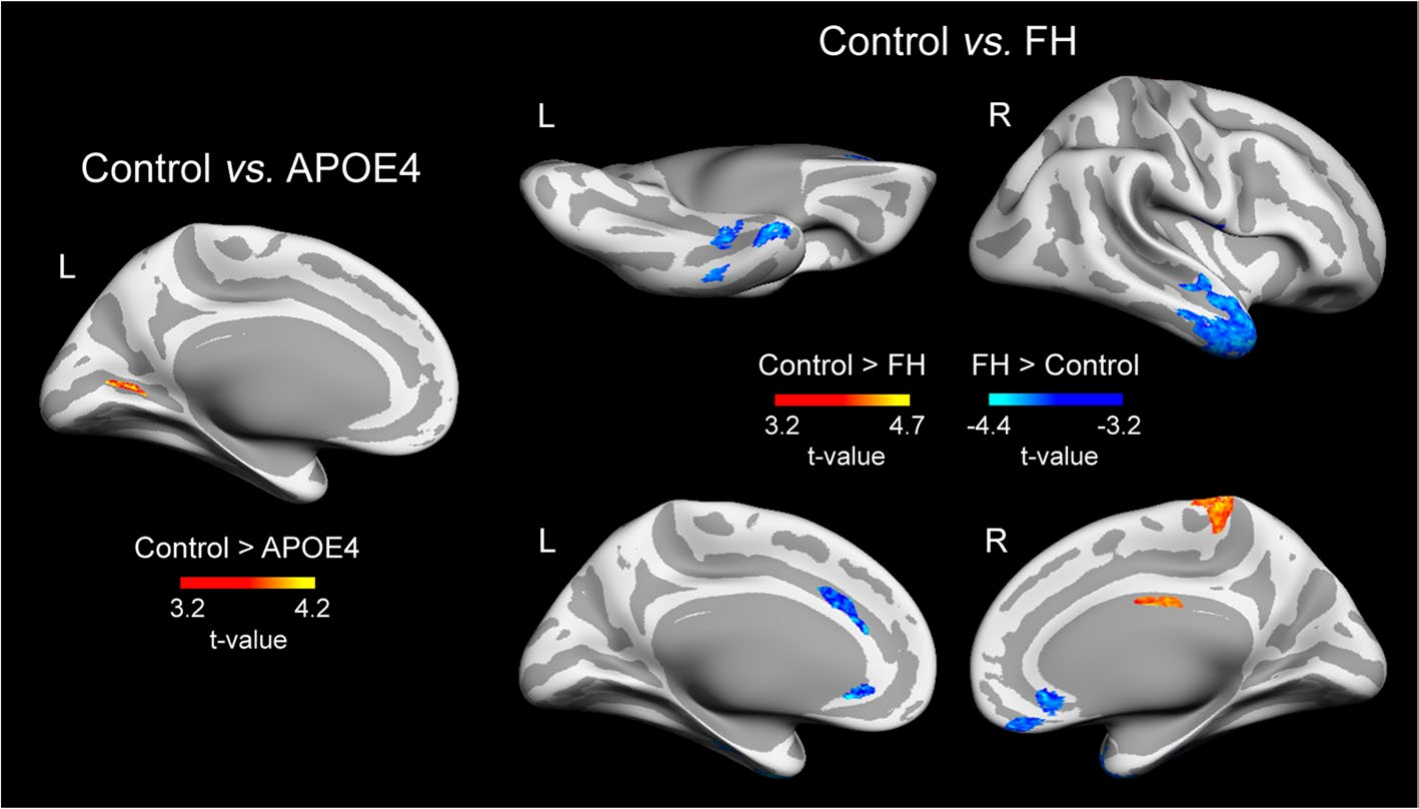
Effects of one single non-modifiable AD risk factor on rT1w/T2w ratio levels. Results were adjusted by age and sex, and corrected for multiple comparisons. Warm and cold scale bars indicate the range of significant *t*-values for the two directions of the statistical contrast. Left (L) and Right (R)

**Fig. 2:**
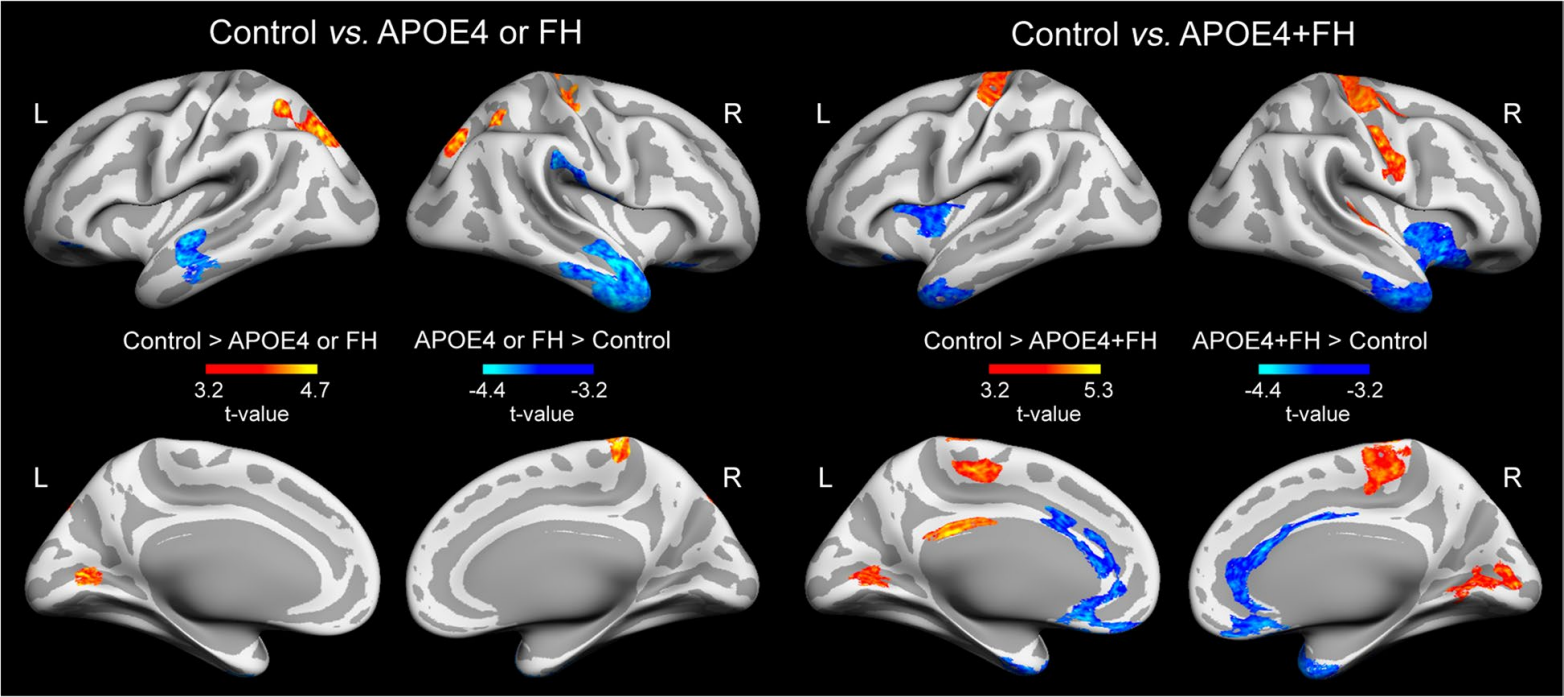
Effects of two non-modifiable AD risk factors on the rT1w/T2w ratio levels. Note that the APOE4 or FH group (left panel) included participants with only one risk factor of AD (either APOE4 or FH), while the APOE4+FH group (right panel) included participants showing the co-occurrence of both AD risk factors (APOE4 and FH). Results were adjusted by age and sex and corrected for multiple comparisons. Warm and cold scale bars indicate the range of significant *t*-values for the two directions of the statistical contrast. Left (L) and Right (R)

Overall, cognitively normal older adults with non-modifiable AD risk factors showed significant changes in rT1w/T2w ratio levels across the cortex that differed as a function of the specific risk factor. These results are detailed in Table 2. Compared to controls, APOE4 carriers showed lower rT1w/T2w ratios in the left lingual gyrus (Fig. 1, left panel), whereas the FH group exhibited lower rT1w/T2w ratios in the right paracentral and posterior cingulate cortex along with higher rT1w/T2w ratios in different regions of the temporal lobe, cingulate cortex, and medial orbitofrontal cortex (Fig. 1, right panel). The group that merged individuals with either APOE4 or FH showed lower rT1w/T2w ratios in superior parietal regions bilaterally, left lingual gyrus, and somatosensory cortex together with higher rT1w/T2w ratios in superior temporal cortex bilaterally, left fusiform, and right supramarginal gyri (Fig. 2, left panel). However, size effects derived from this analysis were consistently lower than those obtained from analysis performed with each AD risk factor separately (see Table 2).

**Table 2:** Group differences in T1w/T2w ratio maps

***Statistical contrast*** Peak location	Extent of change (mm^2^)	MNI coordinates	*F*	*P*	*f*^2^
***Control > APOE4***					
L lingual gyrus	185	− 23 − 66 4	17.3	10^−4^	0.18_M_
***Control > FH***					
R paracentral	271	3 − 38 68	21.9	10^−5^	0.21_M_
R posterior cingulate	61	4 − 4 31	17.2	10^−4^	0.17_M_
***FH > control***					
L inferior temporal	96	− 54 − 28 − 28	16.9	10^−7^	0.16_M_
L entorhinal	195	− 35 − 19 − 29	19.3	10^−6^	0.19_M_
L fusiform gyrus	243	− 30 − 5 − 39	17.3	10^−4^	0.17_M_
L rostral anterior cingulate	72	− 5 29 − 5	14.1	10^−3^	0.14_S_
L caudal anterior cingulate	192	− 11 21 29	13.8	10^−2^	0.13_S_
R middle temporal	2012	48 8 − 37	17.5	10^−7^	0.17_M_
R fusiform gyrus	95	38 − 21 − 29	16.6	10^−6^	0.16_M_
R rostral anterior cingulate	103	3 22 − 8	15.5	10^−5^	0.15_M_
R medial orbitofrontal	158	6 48 − 24	19.3	10^−4^	0.19_M_
R postcentral	100	59 − 11 14	13.6	10^−2^	0.13_S_
***Control > APOE4+FH***					
L isthmus cingulate	213	− 6 − 34 26	27.7	10^−6^	0.41_L_
L precentral	852	− 12 − 25 74	20.5	10^−6^	0.30_M_
L pericalcarine	476	− 21 − 71 7	15.8	10^−5^	0.23_M_
L paracentral	261	− 6 − 24 56	27.3	10^−3^	0.40_L_
R pericalcarine	886	11 − 80 11	20.9	10^−6^	0.31_M_
R paracentral	506	4 − 28 53	27.3	10^−6^	0.40_L_
R precentral	1162	8 − 33 70	25.3	10^−6^	0.37_L_
R precentral	683	48 − 7 28	26.8	10^−5^	0.39_L_
R transverse temporal	433	59 − 9 4	23.5	10^−4^	0.35_L_
***APOE4+FH > control***					
L medial orbitofrontal	1179	− 5 23 − 13	21.2	10^−6^	0.31_M_
L entorhinal	1481	− 24 3 − 39	17.7	10^−6^	0.26_M_
L medial orbitofrontal	119	− 7 29 − 23	24.7	10^−6^	0.36_L_
L insula	674	− 29 21 10	16.9	10^−5^	0.25_M_
L lateral orbitofrontal	136	− 27 11 − 20	16.3	10^−4^	0.24_M_
R lateral orbitofrontal	3907	33 23 − 21	31.6	10^−6^	0.46_L_
R caudal anterior cingulate	807	5 28 18	25.7	10^−6^	0.38_L_
R medial orbitofrontal	317	5 26 − 13	20.6	10^−6^	0.30_M_
***APOE4 > APOE4+FH***					
L pericalcarine	160	− 7 − 90 4	16.1	10^−3^	0.19_M_
L insula	268	− 31 − 27 17	14.1	10^−3^	0.17_M_
L precentral	255	− 42 − 13 34	19.7	10^−2^	0.24_M_
R precentral	258	59 − 2 22	17.8	10^−3^	0.21_M_
R paracentral	220	4 − 28 52	21.2	10^−3^	0.26_M_
***APOE4+FH > APOE4***					
L medial orbitofrontal	264	− 7 35 − 23	27.7	10^−6^	0.33_M_
R medial orbitofrontal	96	3 33 − 22	20.2	10^−5^	0.20_M_
***Control > APOE4 or FH***					
L lingual gyrus	374	− 14 − 77 8	16.2	10^−5^	0.09_S_
L superior parietal	653	− 28 − 55 51	18	10^−4^	0.1_S_
R paracentral	249	3 − 38 68	20.1	10^−6^	0.11_S_
R superior parietal	507	26 − 79 41	20.3	10^−3^	0.12_S_
R precentral	371	26 − 26 52	15.6	10^−2^	0.08_S_
***APOE4 or FH > Control***					
L superior temporal	743	− 51 − 14 − 8	17.4	10^−5^	0.09_S_
L pars orbitalis	118	− 41 36 − 14	13.7	10^−4^	0.07_S_
L fusiform gyrus	122	− 32 − 7 − 41	12.6	10^−3^	0.07_S_
R superior temporal	2641	48 5 − 25	15.1	10^−7^	0.08_S_
R lateral orbitofrontal	166	26 22 − 23	14.3	10^−5^	0.08_S_
R supramarginal	406	55 − 29 34	19	10^−4^	0.1_S_

MNI coordinates correspond to MNI152 standard space. *P*, *P*-value of the cluster; *f*^2^, measure of global effect size. Effect size (*f*^2^): small (S) ≥ 0.02, medium (M) ≥ 0.15, large (L) ≥ 0.35. L/R: left/right

Significant differences in rT1w/T2w ratio levels were much more evident in the group showing the co-occurrence of the two AD risk factors. Thus, the APOE4+FH group showed lower rT1w/T2w ratios in the isthmus cingulate, pericalcarine, precentral, paracentral, and transverse temporal gyrus and higher rT1w/T2w ratios in medial and lateral aspects of the orbitofrontal, entorhinal, and insular cortex compared to controls (Fig. 2, right panel).

**Fig. 3:**
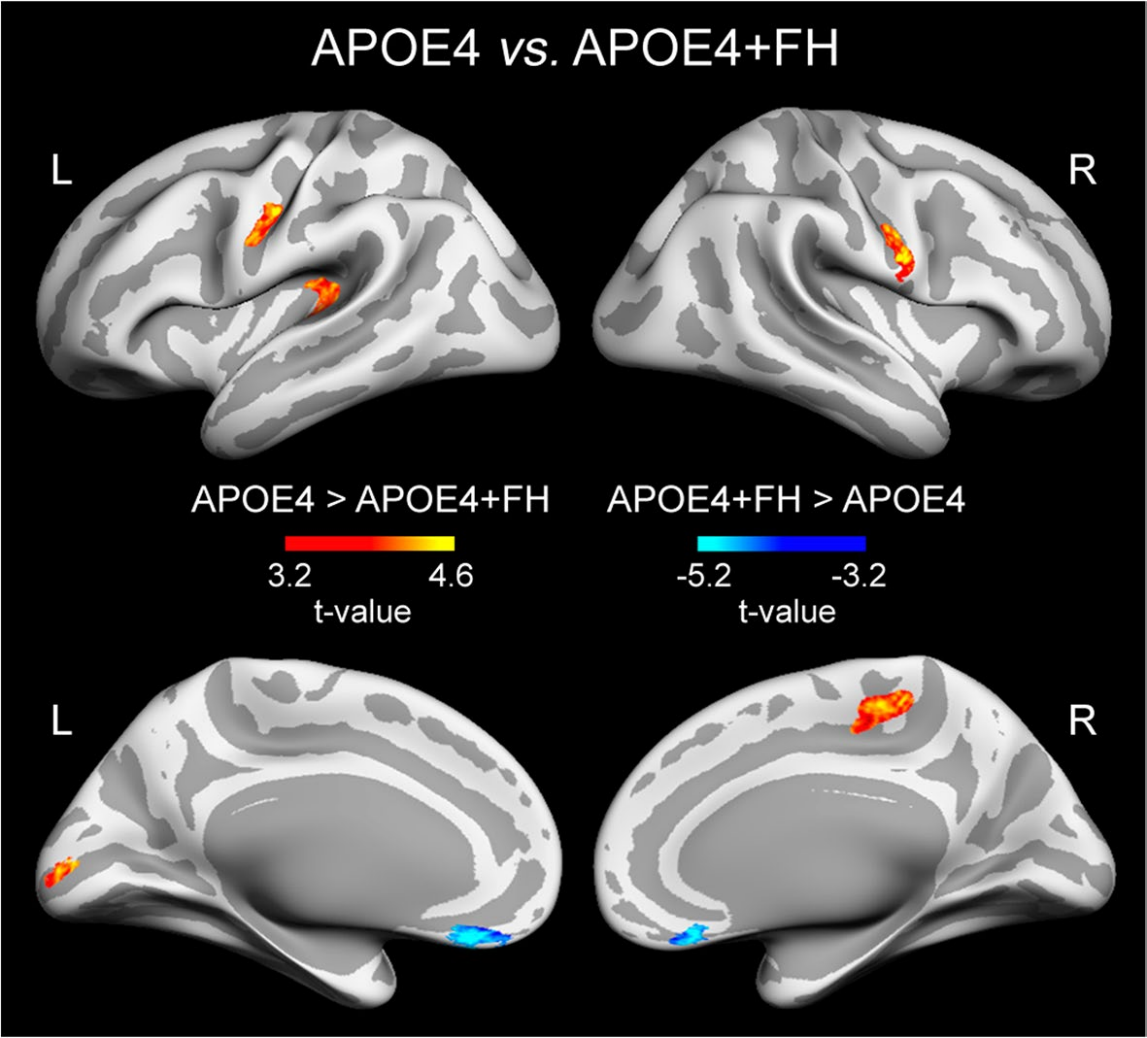
Significant differences in rT1w/T2w ratio levels when comparing the APOE4 and APOE4+FH groups. Results were adjusted by age and sex and corrected for multiple comparisons. Warm and cold scale bars indicate the range of significant *t*-values for the two directions of the statistical contrast. Left (L) and right (R)

rT1w/T2w ratio levels also differed between APOE4+FH and APOE4 groups. Compared to APOE4, older adults with APOE4+FH showed lower rT1w/T2w ratios in precentral, paracentral, pericalcarine, and insular cortex along with higher rT1w/T2w ratios in medial orbitofrontal cortex bilaterally (Fig. 3). In contrast, no significant differences in relative T1w/T2w ratios were found between APOE4+FH and FH groups.

### Effects of non-modifiable AD risk factors on the relationship between T1w/T2w ratio and rs-FC patterns

**Fig. 4:**
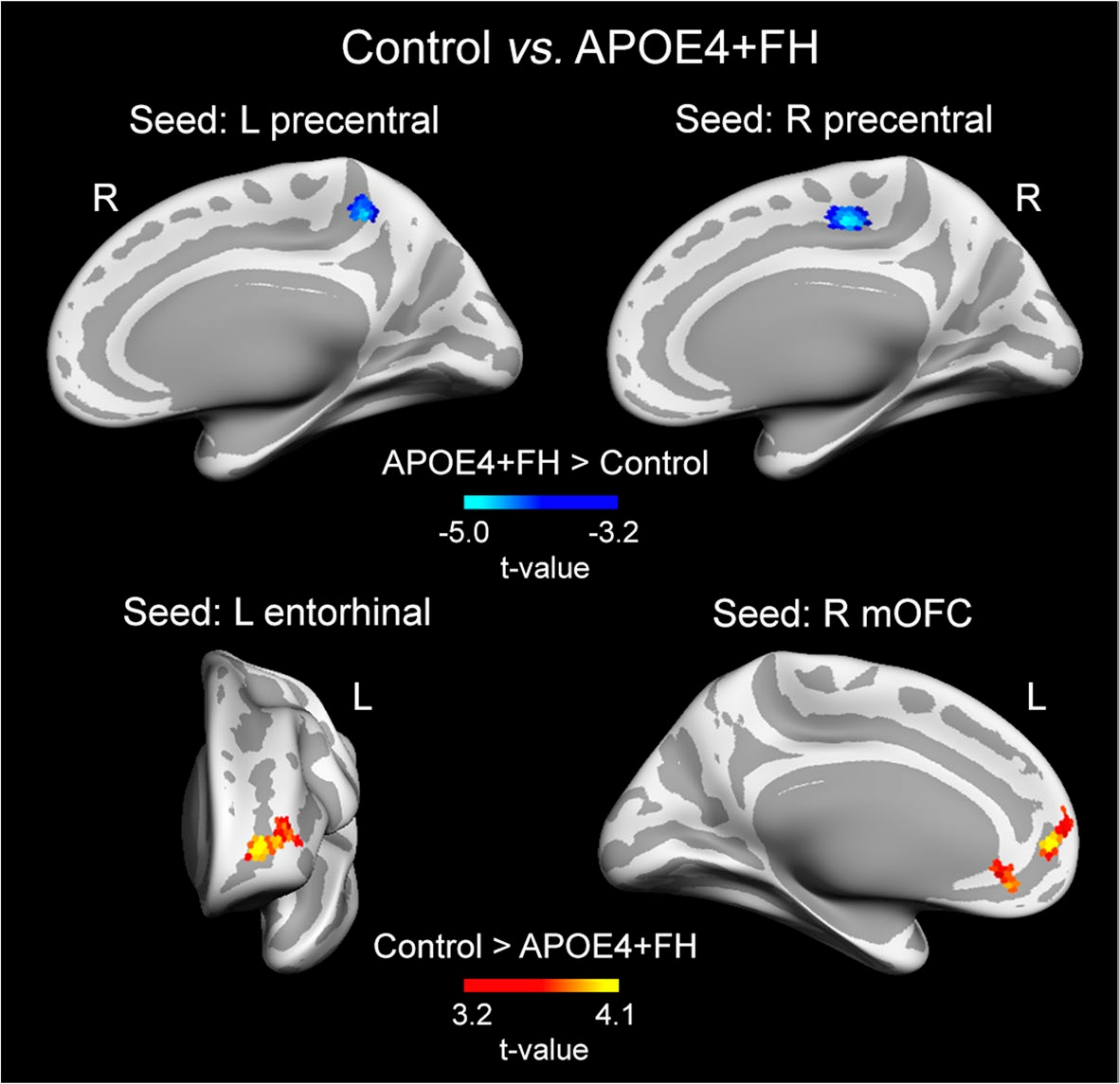
Effects of the co-occurrence of APOE4 and FH of AD (APOE4+FH) on the relationship between rT1w/T2w ratio levels and rs-FC patterns. Upper panel: results obtained with FC seeds that showed lower rT1w/T2w ratios in the APOE4+FH group compared with controls. Analyses showed a positive relationship in the APOE4+FH group and a negative relationship in controls. Bottom panel: results obtained with FC seeds that showed higher rT1w/T2w ratios in the APOE4+FH group compared with controls. The relationship was negative in both groups but it was more negative in the APOE4+FH group than in controls. Analyses were adjusted by age and sex and corrected for multiple comparisons. Warm and cold scale bars indicate the range of significant *t*-values for the direction of the statistical contrast. Left (L) and right (R). mOFC, medial orbitofrontal cortex

Significant group differences in vertex-wise correlation analysis between rs-FC patterns and cortical regions showing significant group differences in rT1w/T2w ratio maps were only evident when participants with APOE4+FH were compared with controls, as derived from group × rT1w/T2w ratio interactions in different rs-FC networks (Table 3). Post hoc analyses revealed that the lower the rT1w/T2w ratios in the left and right precentral gyrus, the lower the FC with the precuneus and paracentral gyrus of the right hemisphere, respectively, in the APOE4+FH group compared to controls (Fig. 4). Additionally, the lower the rT1w/T2w ratios in the left entorhinal and right medial orbitofrontal cortex, the higher the FC with different regions of the left frontal lobe in the APOE4+FH group compared to the control group (Fig. 4).

**Table 3:** Effects of the co-occurrence of APOE4 and FH of AD (APOE4+FH) on the relationship between T1w/T2w ratio values and rs-FC

***FC seed*** Peak location of significant result	Extent of change (mm^2^)	MNI coordinates	*F*	*P*	*f*^2^
***L precentral*** *(lower T1w/T2w ratio in APOE4+FH)*					
R precuneus	138	14 − 44 54	15.5	10^−2^	0.23_M_
***R precentral*** *(lower T1w/T2w ratio in APOE4+FH)*					
R paracentral	162	9 − 21 48	25.4	10^−3^	0.37_L_
***L entorhinal*** *(higher T1w/T2w ratio in APOE4+FH)*					
L rostral middle frontal	435	− 23 53 1	17.2	10^−3^	0.25_M_
***R medial orbitofrontal*** *(higher T1w/T2w ratio in APOE4+FH)*					
L superior frontal	172	− 7 57 9	12.6	10^−2^	0.19_M_
L anterior cingulate	108	− 6 32 − 10	11.3	10^−2^	0.17_M_

MNI coordinates correspond to MNI152 standard space. *P*, *P*-value of the cluster; *f*^2^, measure of global effect size. Effect size (*f*^2^): small (S) ≥ 0.02, medium (M) ≥ 0.15, large (L) ≥ 0.35. L/R: left/right

## Discussion

In this study, we have shown that cognitively unimpaired older adults with non-modifiable AD risk factors have altered tissue integrity in normal-appearing cortical GM, as revealed by variations in rT1w/T2w ratio levels across the cortex. These changes were further accompanied by aberrant rs-FC patterns in individuals showing the co-occurrence of APOE4 and FH of AD. Collectively, these results support the rT1w/T2w ratio as a potential MRI marker for detecting cortical abnormalities in older adults with non-modifiable AD risk factors and open new avenues to monitor changes in cortical tissue integrity associated with these AD risk factors.

Previous research has revealed that normal APOE4 carriers have lower T1w/T2w ratios in WM tracts corroborating the increased vulnerability of WM microstructure in this at-risk population [40]. In the present study, we showed that APOE4 carriers exhibited lower rT1w/T2w ratios in the left lingual gyrus compared to controls. The left lingual gyrus has shown decreased rs-FC patterns in prodromal AD [41] and lower PET activation during an episodic memory task in AD patients [42]. The lower tissue integrity of the left lingual gyrus in normal APOE4 carriers may precede these metabolic and FC deficits, emerging as an early signature of cortical deficits in this high-risk population. Moreover, recent evidence suggests that microglia expressing APOE4 increases the phagocytic uptake of myelin and impairs the ability to degrade internalized myelin [43], linking APOE4 to functional alterations of microglia, a central player in the removal of myelin debris preceding the remyelination process [44].

Our study is the first to examine changes in rT1w/T2w ratio maps associated with a parental history of AD. Similarly to the group showing the co-occurrence of APOE4 and FH, the FH group exhibited higher rT1w/T2w ratio levels in fronto-temporal regions and lower rT1w/T2w ratios in the right paracentral lobule, a cortical hub able to distinguish between individuals at low and high risk of developing AD [45]. Multiple lines of evidence have shown that early Aβ pathology could manifest either as focal myelin loss in AD patients and transgenic mouse models of AD [46, 47] or as an increase in oligodendrogenesis paralleled by thicker myelin sheaths in hippocampal axons of transgenic mouse models of AD [48, 49]. In cognitively normal elderly individuals [50] and AD patients [51], higher T1w/T2w ratio values spread across the temporal lobe, anterior cingulate, and orbitofrontal cortex, regions that have shown to accumulate amyloid deposition before the onset of AD symptoms [52–54]. These late-myelinating cortical regions are particularly susceptible to AD-related neuronal changes and, consequently, more prone to experience myelin breakdown [55]. Alternatively, higher relative T1w/T2w ratio values observed in FH and APOE4+FH groups could act either as a compensatory mechanism for myelin loss manifested in other cortical regions [56] or as a compensatory remyelination affecting targeted cortical regions [57]. The latter is supported by evidence showing that shorter internodes of remyelinated axons affect the speed of axonal conduction [20, 58], which may also contribute for abnormal FC patterns showed by the APOE4+FH group.

While additive effects of APOE4 and FH of AD on brain integrity have been previously described [28, 59–62], the impact of both AD risk factors on cortical microstructure has not been specifically investigated. Our findings suggest that co-occurrence of APOE4 and FH of AD has synergistic effects on tissue integrity in normal-appearing cortical GM, which further lead to aberrant patterns of rs-FC. The precentral gyrus showed abnormal FC with the precuneus and paracentral gyrus of the right hemisphere, which are early myelinating regions involved in AD [53] and affected by non-modifiable AD risk factors [45]. In addition, the precentral gyrus, one of the cortical areas containing the highest density of myelinated axons in the human neocortex [17], exhibited lower relative T1w/T2w ratios bilaterally in individuals with APOE4+FH.

The biological meaning of cortical T1w/T2w ratio maps is currently under debate [63, 64]. MRI-based T1w/T2w ratio maps have been considered as a proxy of myelin content in the cortical GM. This assumption is largely based on the close similitude between T1w/T2w ratio maps and normal cortical myelination patterns [65] across the lifespan [66]. Moreover, signal intensity of T1w and T2w images are directly and inversely proportional to myelin contrast, respectively; thus, the ratio of these images is thought to accentuate the intrinsic contrast of myelin [67]. In addition, magnetization transfer ratio values and R1 mapping, two commonly MRI approaches used to indirectly measure myelin density, have shown to be highly correlated with T1w/T2w ratio maps [68–70]. However, mounting evidence suggests that variations in the T1w/T2w ratio could be influenced by factors other than demyelination [51, 70–78], thus challenging the specificity of the T1w/T2w ratio to myelin content. Therefore, cortical T1w/T2w ratio maps are currently considered as a measure of the microstructural integrity of the normal-appearing cortical GM [79] that is sensible to intracortical myelin content [65].

### Study limitations

This study has some limitations that should be acknowledged. As the biological interpretation of cortical T1w/T2w ratio maps is still imprecise [63, 64], our findings should be interpreted cautiously. More research is clearly needed combining T1w/T2w ratio maps with other imaging modalities and post-mortem studies for a better understanding of microstructural changes in normal-appearing cortical GM. Moreover, the study sample was relatively small and these results should be considered as preliminary and replicated in further experiments. However, it should be noted that effect sizes were above the minimum established, even for the group comparison with the smallest number of participants (i.e., controls vs. APOE4+FH), which showed the largest effect sizes in rT1w/T2w ratio and rs-FC. Additionally, the EPI sequence employed in this study was not corrected for geometric distortions and, therefore, it may suffer from image distortion and signal losses in the most anterior regions of the frontal lobe [80]. Therefore, rs-FC results affecting frontal regions may be partially caused by susceptibility artifacts. Finally, the T1w/T2w ratio cancels RF receive field (B1−) artifacts but does not specifically correct for RF transmit field (B1+) errors, resulting in intensity inhomogeneities in T1w and T2w images that ultimately affect T1w/T2w ratio maps [81]. As B1+ intensity inhomogeneities were not corrected in the present study, we cannot rule out that B1+ errors are affecting our results. Future studies should acquire scans suitable for estimating the B1+ field to correct for transmit field inhomogeneities [82] in order to replicate these findings in datasets unambiguously unaffected by residual B1+ artifacts.

## Conclusions

We provide preliminary evidence of changes in rT1w/T2w ratio maps in older adults carrying either the APOE4 genotype or with a first-degree FH of AD. The magnitude of these changes was most noticeable in individuals showing the co-occurrence of both AD risk factors, which also exhibited abnormalities in rs-FC patterns. These findings indicate that individuals with non-modifiable risk factors of AD have abnormalities in cortical tissue integrity, which may be detected with the T1w/T2w ratio before the onset of cognitive symptoms.

## Supplementary Information

**Additional file 1: Supplementary material Figure 1.** Axial views of T1w, T2w, and EPI images for one representative subject of each group together with their corresponding signal-to-noise ratio (SNR).

## Data Availability

The datasets employed in the current study are available from the corresponding author on reasonable request.

## Abbreviations

AD: Alzheimer’s disease
APOE4: Apolipoprotein E ɛ4 allele
BOLD: Blood-oxygen-level-dependent
CSF: Cerebrospinal fluid
EPI: Single-shot echo-planar
FH: First-degree family history of AD
GM: Gray matter
rs-FC: resting state functional connectivity
WM: White matter
