# Supplementary material for: Effects of non-modifiable risk factors of Alzheimer’s disease on intracortical myelin content
Source: Alzheimers Res Ther. 2022 Dec 31;14:202. doi: 10.1186/s13195-022-01152-y (PMC9805254; doi:10.1186/s13195-022-01152-y)
Supplement: Supplementary file 1 — Additional file 1: Supplementary material Figure 1. Axial views of T1w, T2w, and EPI images for one representative subject of each group together with their corresponding signal-to-noise ratio (SNR). [file 13195_2022_1152_MOESM1_ESM.docx]

**Supplementary Material**

**Effects of non-modifiable risk factors of Alzheimer’s disease on intracortical myelin content**

Marina Fernandez-Alvarez^1,2^, Mercedes Atienza^1,2^, Jose L. Cantero^1,2*^

**Supplementary material, Figure 1**


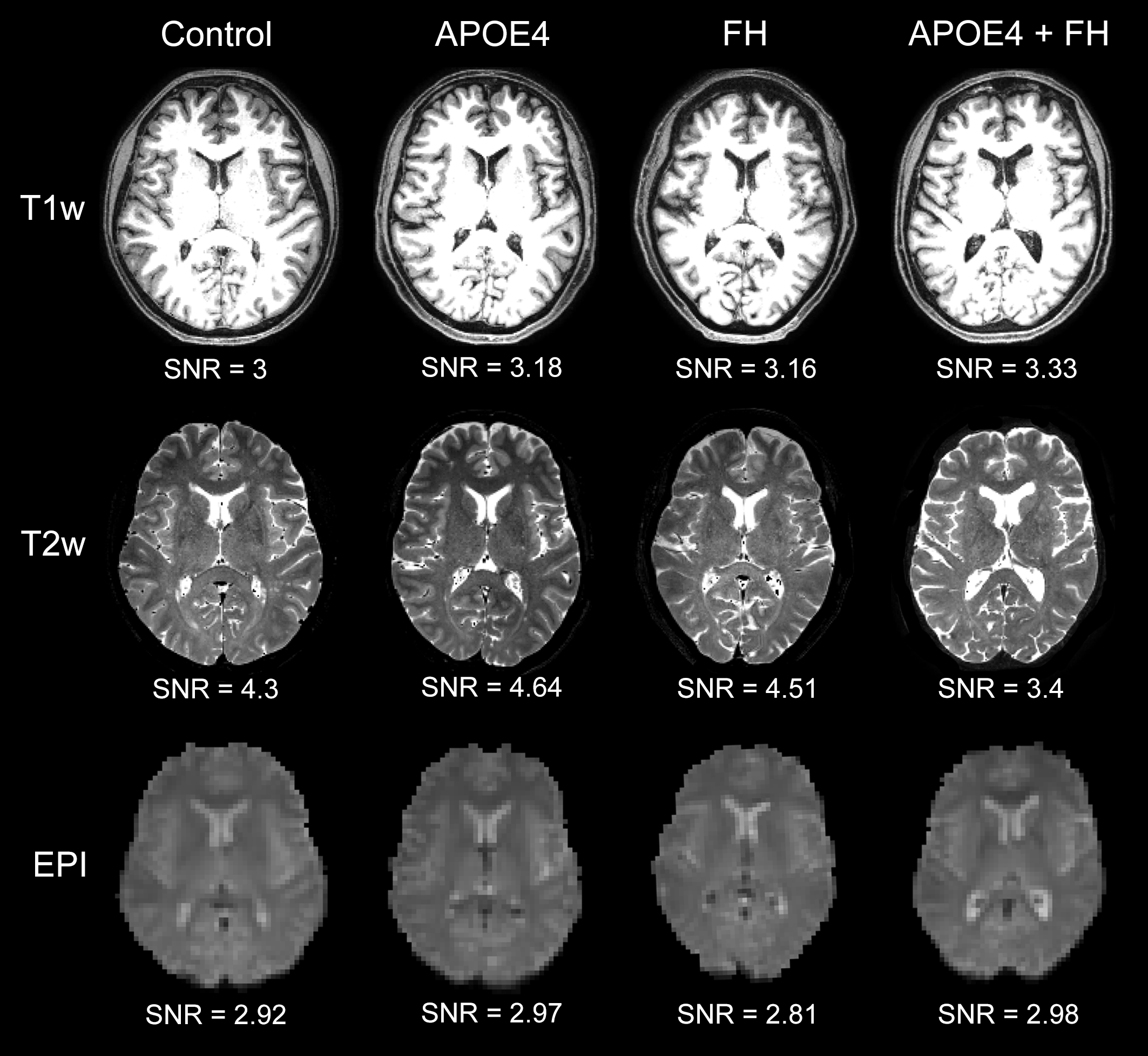


Axial views of T1w, T2w, and EPI images for one representative subject of each group together with their corresponding signal-to-noise ratio (SNR). The SNR for T1w and T2w scans was calculated as the mean divided by the standard deviation of voxel intensities within a gray matter (GM) mask obtained from the Freesurfer aparc+aseg segmentation atlas. This GM mask included the complete cerebral and cerebellar volume and pons. The spatial SNR was similarly computed for the mean EPI map.
